# Imaging of the superficial white matter in health and disease

**DOI:** 10.1162/imag_a_00221

**Published:** 2024-07-22

**Authors:** Peter C. Van Dyken, Ali R. Khan, Lena Palaniyappan

**Affiliations:** Neuroscience Graduate Program, Schulich School of Medicine and Dentistry, Western University, London, ON, Canada; Robarts Research Institute, Western University, London, ON, Canada; Department of Medical Biophysics, Schulich School of Medicine and Dentistry, Western University, London, ON, Canada; Department of Psychiatry, Douglas Mental Health University Institute, McGill University, Verdun, QC, Canada; Department of Psychiatry, Schulich School of Medicine and Dentistry, Western University, London, ON, Canada

**Keywords:** superficial white matter, magnetic resonance imaging, tractography, development, disease, cortical connectivity

## Abstract

The superficial white matter, the layer of white matter immediately deep to the cortical grey matter, is a highly complex, heterogeneous tissue region comprising dense meshes of neural fibres, a robust population of interstitial neurons, and ongoing glial activity and myelination. It originates from the histologically distinct, developmentally vital subplate in the foetal brain, maintains thalamo-cortical connections throughout adult life, and is a necessary passage for all axons passing between the grey and white matter. Despite these features, the superficial white matter is among the most poorly understood regions of the brain, in part due to its complex makeup and the resulting difficulty of its study. In this review, we present our current knowledge of superficial white matter (SWM) anatomy, development, and response to disease. We discuss the unique challenges encountered in the neuroimaging of this region, including the lack of standard definition and the non-specificity of neuroimaging markers amplified by the complexity of the tissue. We discuss recent innovations and offer potential pathways forward.

## 1 Introduction

**Fig. 1.**
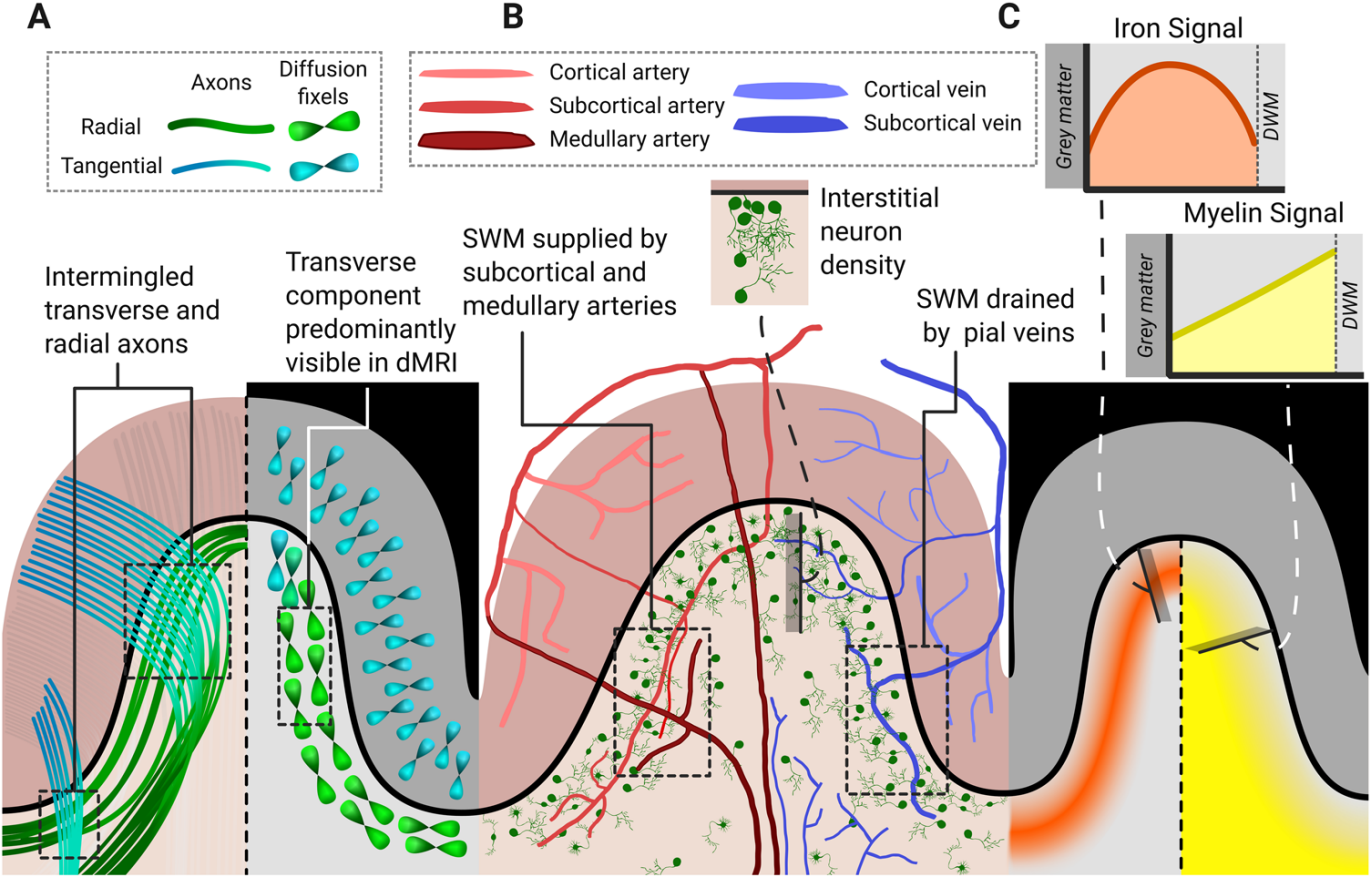
Schematic illustrating basic features of the superficial white matter (SWM). (A) A dense mesh of fibres runs both tangentially along the grey matter–white matter interface and radially into the grey matter (GM) (Cottaar et al., 2018;Reveley et al., 2015;K. Schilling et al., 2018). (B) White matter (WM) interstitial neurons are represented in green. Cell density decreases deeper in the WM (Sedmak & Judaš, 2021). SWM supplied by both subcortical and medullary arteries. Cortex is supplied only by cortical arteries; deep WM (DWM) is supplied only by medullary arteries (Smirnov et al., 2021). Both SWM and cortex are drained by pial veins. DWM is drained by medullary veins (San Millán Ruíz et al., 2009). (C) A band of increased iron signal runs along the SWM. This colocalizes with a linear reduction in myelin signal stretching from the DWM to the SWM (Kirilina et al., 2020;Lee et al., 2023).

The superficial white matter (SWM) refers to the layer of white matter immediately deep to the cortical grey matter. Compared with the relative structural simplicity of the further underlying deep white matter (DWM), the SWM is a complex integration of axonal fascicles, subcortical neurons, glial cells, and vasculature. A dense mesh of axons occupies the layer, amalgamating short, locally connecting fibres with the terminations of long fascicles projecting from across the cortex (Fig. 1A). These features arise from the developmental origins of the SWM and its adjacency to the grey matter and distinguish it from the DWM across the lifespan in health and disease.

In the last two decades, the SWM has received focused attention from neuroimaging studies, quantifying the effects of development and disease on myelin, iron, cytoarchitecture, and microstructural complexity as interpreted with diffusion magnetic resonance imaging (dMRI). Considerable effort has also been dedicated to mapping the complex cortical connectivity mediated by the short association fibres (SAFs) endemic to the layer. However, the same tissue complexity driving this considerable research interest in the SWM also stymies the interpretation of traditional neuroimaging analyses, necessitating a careful, informed approach to experimental planning and interpretation.

In this review, we will outline the microstructural factors distinguishing the SWM from the DWM. With this context, we will summarize the currently recognized short and medium range cortical connections delineated by dissection and neuroimaging and synthesize recent investigations of myelin, iron, and diffusivity across early development, ageing, and disease, drawing on both neuroimaging and histological explorations. Special focus will be paid to interpretive limitations when neuroimaging the SWM. Above all, we illustrate and emphasize the importance of taking a multimodal approach, complementing imaging with ground-truth pathological and histological data, and we highlight exemplary studies embracing these designs.

This work was conducted as a narrative review within a scoping framework (Munn et al., 2018) to identify and analyze the key gaps in neuroimaging research on SWM. Studies were obtained via searches on PubMed via terms including “superficial white matter”, “SWM”, “U-fibers”, “short association fibres”, “SAF”, “tractography”, “Klingler’s dissection”, “schizophrenia”, “Alzheimer’s Disease”, “histology”, “development”, and “aging”. Other miscellaneous terms were used for more focused searches as needed for particular sections. Papers were also included based on citations, both works cited by and works citing seminal sources. The search was performed by the first author (P.V.D.), with the included literature individually appraised by the last author (L.P.). We anticipate this review influencing primary research studies on white matter as well as future systematic searches focused on more specific questions and results from specific populations.

## 2 Cytoarchitecture of the Superficial White Matter

### 2.1 Development

The SWM arises from the remnants of the foetal subplate, the developmental layer immediately deep to the precursor of the grey matter, the cortical plate (Judaš, Sedmak, & Pletikos, 2010). In humans, the subplate forms in the 12th–13th week of gestation, emerging from the deep aspect of the cortical plate. Neurons settled in this region following their migration from the ventricular zone are displaced by incoming axonal growth and spread deep into the brain across the subplate (Duque et al., 2016). The subplate continues to grow and expand throughout the second trimester, reaching its maximal size of 4 times the cortical plate width at 20 weeks. Throughout gestation, the subplate hosts a complex ecosystem of axonal outgrowth, synaptogenesis, migrating neurons, and developing glia, the details of which have been extensively reviewed byKostović et al. (2019). New neurons continue to migrate through the subplate on their journey from the ventricular zone to the cortical plate. Thalamocortical fibres (Burkhalter et al., 1993), then callosal fibres, then corticocortical fibres progressively traverse the subplate and form transient synaptic connections, the beginnings of cortical connectivity. These synapses are replaced with cortical plate synapses beyond the 24th week of gestation. The earliest evidence of myelination is observed at 28 weeks gestation (Counsell et al., 2002), beginning a process that will continue into adolescence. At around 32 weeks of gestation (Kostović & Rakic, 1990;Kostović et al., 2019), cortical layer VI begins to form and the subplate starts to dissolve, giving way to the*centrum semiovale*and the SAFs of the SWM (Kostović & Rakic, 1980). Dissolution proceeds outward toward the cortex. SAFs, accordingly, grow from the bottom of sulcal fundi toward gyral crowns (Kostović et al., 2014). The entire process continues until well after birth, and regions with a thicker mid-gestation subplate, such as the frontal lobe, take the longest to mature. For instance, the subplate disappears from the pre- and post-central gyri and occipital lobe by 13 post-natal months, but is still present in the pre-frontal cortex (PFC), likely for a few more months (Kostović et al., 2014). Remaining cells in the subplate become white matter interstitial neurons (WMINs) (Chun & Shatz, 1989;Kostović & Rakic, 1980) surrounded by the axons of the SWM (Kostović et al., 2014).

### 2.2 Cellular composition

#### 2.2.1 Neurons

The healthy, mature SWM hosts a rich ecosystem of WMINs, which consistently occupy healthy cortical architecture across the lifespan (Fig. 1B) (Sedmak & Judaš, 2021;Suarez-Sola et al., 2009). With an average density of 1200 neurons per mm^3^(Sedmak & Judaš, 2019), they comprise an estimated 3% of all neurons in the cortex (Sedmak & Judaš, 2021). WMIN density is highest at the grey matter–white matter interface (GMWMI), decreasing with cortical depth, with studies reporting four (García-Marín et al., 2010) to five (Mortazavi et al., 2017) times the number of cells in the SWM than the DWM. Density within the SWM is roughly twice as high at the gyral crowns than at the sulcal depths, as determined both in rhesus monkeys (Mortazavi et al., 2017) and chimpanzees (Swiegers et al., 2021). Regional variations also occur. A study of rhesus monkeys found higher WMIN density in the parietal and temporal lobes than in the medial superior frontal gyrus (SFG) and central sulcus (Mortazavi et al., 2017). This regional distribution has generally been confirmed in humans, although some discrepancy remains between studies. The dorsolateral PFC and orbital pole have been shown to have high WMIN density, while occipital lobe/visual areas and the cingulate gyrus have been alternately shown to have relatively high and low densities (García-Marín et al., 2010;Sedmak & Judaš, 2019). Differences in these estimates likely relate to differing methods of segmenting the SWM, as discussed in detail bySedmak and Judaš (2019).

Once established, WMIN populations remain stable (García-Marín et al., 2010), with only limited evidence suggesting a decline of density in the PFC in human ageing (Meencke, 1983). Observations from rhesus monkeys found no evidence of a change in cell density across the lifespan but did observe a reduction in soma size in all regions except the temporal lobe (Mortazavi et al., 2016).

WMINs have a functional and morphological diversity similar to that observed in the grey matter. Like grey matter neurons, they are immunoreactive to NeuN (Meyer et al., 1992;Sarnat et al., 2018;Sedmak & Judaš, 2019) and can be summarily divided into excitatory, glutamatergic, pyramidal neurons, and inhibitory, GABAergic interneurons. The pyramidal neurons are morphologically indistinguishable from those in layer VI of the cortex (Meyer et al., 1992) and include unipolar, bipolar, and multipolar cells, depending on the region (Zouridakis et al., 2023). GABAergic neurons have been found expressing a wide variety of markers, including calcium-binding proteins such as calretinin and parvalbumin; cholinoceptive markers such as the M2-muscarinic receptor and acetylcholinesterase (Smiley et al., 1998;Zouridakis et al., 2023); and nitric oxide synthase, identified via nicotinamide adenine dinucleotide phosphate diaphorase (NADPHd). Together these markers reflect a wide heterogeneity of cell types, including cholinoceptive neurons, calbindin-positive neurons, and nitrinergic neurons, all distinct populations (Zouridakis et al., 2023). Interestingly, up to 80% of all NADPHd-positive neurons in the cortex are WMINs (Fischer & Kuljis, 1994;Judaš, Sedmak, Pletikos, & Jovanov-Milošević, 2010;Kowall & Beal, 1988;Norris et al., 1996). Most of these nitrinergic neurons are cholinoceptive, suggesting they may be a relay for cholinergic regulation of nitric oxide (Smiley et al., 1998). Axonal contact between them and blood vessels has been observed (Zouridakis et al., 2023), and given nitric oxide is a potent vasodilator, they may be important modulators of cerebral blood flow (Suarez-Sola et al., 2009).

However, the actual functions of WMINs are largely unstudied and speculative. In addition to their interactions with blood vessels, they form extensive interactions with both the cortex and thalamus (Clancy et al., 2001;Fischer & Kuljis, 1994;García-Marín et al., 2010;Sarnat et al., 2018;Valverde & Facal-Valverde, 1988) and fully participate in cortical networks (Torres-Reveron & Friedlander, 2007). They may thus regulate the flow of information across the SWM, given their position at the juncture of the DWM and grey matter (Sedmak & Judaš, 2021), a hypothesis originally argued byColombo (2018).

#### 2.2.2 Glia

Unlike WMINs, which are a peculiar feature of the SWM, very little work has investigated the properties of glial cells specific to the SWM. Nevertheless, some basic characteristics can be noted. Fibrous astrocytes populate the SWM just as in the rest of the white matter (Oberheim et al., 2009), but protoplasmic astrocytes, which are typically observed in the grey matter with extensive synaptic contacts (Zisis et al., 2021), have not been observed, despite the presence of WMINs (Oberheim et al., 2009). Beyond these basic glial types, varicose projection astrocytes, a subtype specific to humans and great apes, have been observed both in the lowest layers of the grey matter (V-VI) and in the SWM (Falcone et al., 2022). Finally, Miller and Raff’s original work delineating fibrous and protoplasmic astrocytes described an intermediary phenotype at the edge of the optic nerve, suggesting such “boundary” astrocytes may play both grey matter- and white matter-related roles (Butt et al., 2014;Miller & Raff, 1984).

Oligodendrocytes regulate the myelination of neurons. They have a particularly high density in the SWM—more so than the overlying grey matter (Z. He et al., 2017)—corresponding with the relatively late myelination of the layer’s dense axonal tracts (seeSection 4.1.3). Iron, a key factor for myelination, colocalizes with these oligodendrocytes, resulting in an intense band of iron-specific signal viewable on imaging (Kirilina et al., 2020) (Fig. 1C). It is not known whether SWM oligodendrocytes more closely resemble those of the grey matter, which have small cell bodies, support numerous axonal interactions with fewer myelin layers, and mature relatively late, or those of the DWM, which have larger cell bodies, and support fewer axonal interactions with more layers (Butt & Berry, 2000;Haroutunian et al., 2014;Osanai et al., 2022). The high axonal density suggests the former, but this must be confirmed by region-specific evaluation. Nevertheless, the high oligodendrocyte density suggests a unique dependency within the SWM and, possibly, a corresponding vulnerability to myelin-related disorders.

Finally, microglia are the resident immune cells within the otherwise immunoprotected cortical parenchyma. Microglial cell counts are higher throughout the white matter than the grey matter (Mittelbronn et al., 2001), and this appears to hold true in the SWM (Taipa et al., 2017). Microglia are known to play a role in myelination (Borst et al., 2021), but this interaction has not been particularly studied in SWM.

### 2.3 Vascular organization

The SWM can be further distinguished by its vascular supply (Fig. 1B). Endoparenchymal arteries supplying the cortex and white matter arise from the pial vascular network and extend toward the ventricles. These can be classified according to their length: cortical arteries are restricted to the cortex, subcortical arteries extend into the SWM, and medullary arteries travel into the DWM as far as the lateral ventricles. The cortex and DWM are both supplied solely by their corresponding artery class. The SWM, in contrast, receives supply both from subcortical arteries and the proximal branches of the medullary arteries. This dual supply makes the SWM more resilient to blood flow-related pathology, such as lacunar infarcts and small vessel disease (Riley et al., 2018;Smirnov et al., 2021).

Venous drainage of the SWM occurs via the superficial venous system, which combines with the cortical circulation and collects into the pial veins. The DWM drains via the deep medullary veins, which collect at the ventricles into the subependymal veins (San Millán Ruíz et al., 2009).

This separated circulation between the two white matter layers corresponds with markedly different activity patterns as assessed by functional magnetic resonance imaging (fMRI). The blood oxygen level dependent (BOLD) signal (which represents a local increase in blood flow in response to increased neural activity) observed in the DWM is qualitatively different than that of the grey matter. Geometrically, its peak is shorter, narrower, and has a smaller integral area; the undershoot following the peak is shorter; and the peak is preceded by a dip. Deeper dips are associated with narrower, more delayed peaks (K. G. Schilling, Li, et al., 2022). Furthermore, the timing of the DWM BOLD response is delayed relative to the grey matter by as much as twofold (M. Li et al., 2019). Functional correlations between grey matter and DWM are maximized with a 4–6 second delay incorporated into the DWM signal (M. Li et al., 2020). However, none of these features manifest in the SWM. Profiling the BOLD response along various white matter tracts, segments nearest to the cortex consistently show a response more similar to that seen in the grey matter (K. G. Schilling, Li, et al., 2022). Task-linked and internal functional connectivity is not delayed but synchronized with the grey matter (M. Li et al., 2019,^2020^). These differences are reflected in experiments clustering the white matter based on functional activity, which consistently split the region into deep, middle, and superficial clusters (Jiang, Luo, et al., 2019;Jiang, Song, et al., 2019;J. Li et al., 2022;Peer et al., 2017).

These observations suggest that functional activity in the SWM may be statistically dependent on the functional activity of the overlying grey matter. In the data fromPeer et al. (2017), many of the superficial clusters are strongly correlated with immediately adjacent, independently clustered grey matter regions, although they did not explicitly test this association. On the other hand, such an observation was not evident in more recent work fromP. Wang et al. (2022), but again, the association was not explicitly tested. Nevertheless, statistical linkage between adjacent grey matter and white matter can be predicted both from their common circulatory drainage (Huck et al., 2023) and from the functional linkage expected between a grey matter region and the axons directly connected to it. The BOLD signal is broadly homogeneous along individual white matter fascicles (Ding et al., 2013,^2016^;Mezer et al., 2009;Peer et al., 2017), and fascicular functional activity is related to task-based neurological activation (M. Li et al., 2019). Therefore, one might reasonably expect strong correlations between SWM fibres and their connected regions. Finally, the putative role of WMINs in the regulation of the haemodynamic response, described above, also predicts a functional linkage between grey matter and adjacent SWM. As an important caveat, however, the proximity of the SWM to the grey matter increases its susceptibility to partial volume effects. These correlations might thus be more explainable by imprecise sampling than true physiological linkage.

## 3 Fascicular Organization

### 3.1 Overview

Perhaps the most salient feature of the SWM is the dense mesh of axons running transverse to the GMWMI and radially penetrating the grey matter (Fig. 1A) (K. Schilling et al., 2018). While cortical axons of all lengths must pass through the SWM, the transverse component of the mesh largely comprises medium association fibres (MAFs) and SAFs. MAFs include the frontal aslant tract (FAT), vertical occipital fasciculus, sledge runner, and temporo-parietal connection; they are usually intra-lobar but may connect adjacent lobes. SAFs, typically less than 40 mm long, connect immediately adjacent gyri (inter-gyral), or the gyral walls within a single gyrus (intra-gyral). They are nearly ubiquitous, meaning any two adjacent gyri selected will have white matter tracts connecting them (Monroy-Sosa et al., 2020;Shinohara et al., 2020). Their density, however, varies significantly across the brain, allowing the delineation of robust, reproducible SAF bundles. Such bundles are often called “U-fibres”, due to their tight, concave curve. MAFs and explicitly delineated SAFs are tabulated inTable 1.

**Table 1.** Medium association fascicles and short association fascicles in the human brain.

Tract	Course	Tractography studies	Dissection studies
**Frontal Aslant Tract (FAT)**	Anterior supplementary and pre-supplementary motor of superior frontal gyrus to pars triangularis, pars opercularis, ventral aspect of pre-central gyrus	( Bozkurt et al., 2017 ; Catani et al., 2012 ; Catena Baudo et al., 2023 ; Pascual-Diaz et al., 2020 ; Rojkova et al., 2016 ; Varriano et al., 2020 )	( Bozkurt et al., 2016 , 2017 ; Catani et al., 2012 ; Catena Baudo et al., 2023 ; Monroy-Sosa et al., 2020 ; Pascual-Diaz et al., 2020 ; Vergani, Lacerda, et al., 2014 )
Fronto-orbito-polar	Posterior orbital gyrus to anterior orbital gyrus and ventromedial orbital pole	( Catani et al., 2012 ; Rojkova et al., 2016 )	( Catani et al., 2012 )
Fronto-marginal	Medial to lateral regions of fronto-polar cortex, beneath fronto-marginal sulcus	( Catani et al., 2012 ; Rojkova et al., 2016 )	( Catani et al., 2012 )
Hand knob tracts	Pre- to post-central gyrus in the hand knob region of central sulcus	( Catani et al., 2012 ; Pron et al., 2021 ; Rojkova et al., 2016 ; Simone et al., 2021 )	( Catani et al., 2012 ; Vergani, Lacerda, et al., 2014 )
Face tracts	Pre- to post-central gyrus in the ventral aspect of central sulcus	( Catani et al., 2012 ; Pron et al., 2021 ; Rojkova et al., 2016 )	
Paracentral tracts	Pre- to post-central gyrus in the dorsal aspect of central sulcus	( Catani et al., 2012 ; Pron et al., 2021 ; Rojkova et al., 2016 )	
Fronto-insular tracts (FIT)	Inferior frontal gyrus and pre-central gyrus, around the peri-insular sulcus, to the insula	( Catani et al., 2012 ; Rojkova et al., 2016 )	( Catani et al., 2012 )
Frontal Longitudinal System (FLS) Superior	Pre-central gyrus to ventral part of superior frontal gyrus and dorsal part of middle frontal gyrus	( Bozkurt et al., 2017 ; Catani et al., 2012 ; Rojkova et al., 2016 )	( Bozkurt et al., 2016 , 2017 ; Briggs et al., 2021 ; Catani et al., 2012 ; Maldonado et al., 2012 ; Vergani, Lacerda, et al., 2014 )
FLS Inferior	Pre-central gyrus to ventral part of middle frontal gyrus and dorsal part of inferior frontal gyrus	( Catani et al., 2012 ; Rojkova et al., 2016 )	( Briggs et al., 2019 , 2021 ; Catani et al., 2012 )
Suppl. Motor Area-Cingulate Gryus	Supplementary motor area to cingulate gyrus		( Vergani, Lacerda, et al., 2014 )
Precuneus—Cuneus	Precuneus to cuneus		( Vergani, Mahmood, et al., 2014 )
*Stratum Calcarinum*	Upper to lower banks of calcarine fissure	( Bugain et al., 2021 )	( Vergani, Mahmood, et al., 2014 )
**Medial Occipital Longitudinal Tract (MOLT) (sledge runner)**	Inferior aspect of cuneus to superior/anterior aspect of lingual gyrus, further extending to the posterior parahippocampal gyrus	( Beyh et al., 2022 ; Bugain et al., 2021 ; Koutsarnakis et al., 2019 )	( Baydin et al., 2017 ; Koutsarnakis et al., 2019 ; Vergani, Mahmood, et al., 2014 )
*Stratum Proprium Cunei*	Superior to inferior aspect of cuneus	( Bugain et al., 2021 )	( Vergani, Mahmood, et al., 2014 )
*Stratum Verticale Convexitatis*	Between occipital gyri under the superior, middle, and inferior occipital sulci		( Vergani, Mahmood, et al., 2014 )
*Stratum Profundum Convexitatis*	Superior to inferior aspect of occipital lobe		( Vergani, Mahmood, et al., 2014 )
*Stratum Sagittale*	Occipital to temporal lobes, wrapping around the occipital ventricular lobe		( Vergani, Mahmood, et al., 2014 )
**Vertical Occipital Fasciculus (VOF)**	Occipito-temporal sulcus, ascending to lateral superior occipital lobe and posterior angular gyrus	( Bugain et al., 2021 ; Bullock et al., 2019 ; Jitsuishi et al., 2020 ; Palejwala et al., 2020 ; Petit et al., 2023 ; Takemura et al., 2016 , 2017 ; Y. Wu et al., 2016 ; Yeatman et al., 2013 , 2014 )	( Jitsuishi et al., 2020 ; Palejwala et al., 2020 ; Petit et al., 2023 ; Y. Wu et al., 2016 )
Transverse Fasciculus of the lingual lobule of Vialet	Inferior gyri of calcarine fissure to infero-lateral aspects of occipital lobe	( Bugain et al., 2021 )	
**Temporo-Parietal Connection**	Inferior and middle temporal gyri, fuisiform gyrus, inferior occipital lobe to superior parietal lobe	( Bullock et al., 2019 ; Y. Wu et al., 2016 )	
Parietal Inferior-to-Superior tract (PIST)	Superior parietal lobule to supramarginal and angular gyri	( Burks et al., 2017 ; Catani et al., 2017 ; Petit et al., 2023 )	( Burks et al., 2017 ; Catani et al., 2017 ; Petit et al., 2023 )
Parietal Inferior-to-Post-central (PIP)	Suppramarginal and angulary gyri to post-central gyrus	( Catani et al., 2017 ; Petit et al., 2023 )	( Catani et al., 2017 ; Petit et al., 2023 )
Parietal Superior-to-Post-central (PSP)	Superior parietal lobule to post-central gyrus	( Catani et al., 2017 )	( Catani et al., 2017 ; Maldonado et al., 2012 )
Parietal Angular-to-Supramarginal (PAS)	Angular gyrus to supra-marginal gyrus	( Burks et al., 2017 ; Catani et al., 2017 ; Petit et al., 2023 )	( Burks et al., 2017 ; Catani et al., 2017 ; Petit et al., 2023 )
Parietal Intra-gyral (PIG) of the Supramarginal gyrus	Intra-gyral U-fibres within supra-marginal gyrus	( Catani et al., 2017 )	( Catani et al., 2017 )
PIG of the Precuneus	Intra-gyral U-fibres within precuneus gyrus	( Catani et al., 2017 ; Tanglay et al., 2022 )	( Catani et al., 2017 ; Tanglay et al., 2022 )
PIG of the Superior Parietal Lobe (SPL)	Intra-gyral U-fibres within superior parietal lobule	( Catani et al., 2017 )	( Catani et al., 2017 )
Precuneus-SPL	Precuneus to superior parietal lobule	( Tanglay et al., 2022 )	( Maldonado et al., 2012 ; Tanglay et al., 2022 )
Intra-gyral Insular Cortex	Intra-gyral U-fibres within the insula	( Nachtergaele et al., 2019 )	( Nachtergaele et al., 2019 )
Insular Cortex—Medial Temporal Lobe	Insula to medial temporal lobe	( Nachtergaele et al., 2019 )	( Nachtergaele et al., 2019 )
Angular Gyrus (AG) —Precuneus	Angular gyrus to precuneus	( Petit et al., 2023 )	( Petit et al., 2023 )
AG—Occipital Lobe (all gyri)	Angular gyrus to the superior, middle, and inferior occipital gyri	( Petit et al., 2023 )	( Petit et al., 2023 )
AG—Temporal Lobe (superior and middle)	Angular gyrus to superior and middle temporal gyri	( Petit et al., 2023 )	( Petit et al., 2023 )
AG—Pre-central Gyrus	Angular gyrus to pre-central gyrus	( Petit et al., 2023 )	( Petit et al., 2023 )
Lingual Gyrus—Fusiform Gyrus	Lingual gyrus to fusiform gyrus	( Palejwala et al., 2020 )	( Palejwala et al., 2020 )
Fusiform Gyrus—Inferior Occipital Gyrus	Fusiform gyrus to inferior occipital gyrus	( Palejwala et al., 2020 )	( Palejwala et al., 2020 )

Tract names are specified according to the nomenclature most used in the literature (leading to inconsistent naming schemes across the lobes). Tracts with no specific name are labelled according to their termination points. FLS = Frontal Longitudinal System; PIG = Parietal Intra-gyral; SPL = Superior Parietal Lobe; AG = Angular Gyrus.

### 3.2 Methods of studying short association fibres

Because of the difficulty executing tract-tracing and other invasive paradigms enjoyed in animal models (Jbabdi et al., 2015), two methods have been of practical utility for exploring long range connectivity in humans. Pathological dissection on post-mortem samples has been used for over a century (Klingler, 1935;Yeatman et al., 2014) in the classification of white matter fascicles. More recently, tractography via dMRI has taken its place as the primary tool of choice (Catani et al., 2012;Guevara et al., 2020;Jbabdi et al., 2015) (for primers on tractography, seeMukherjee et al. (2008)andJbabdi & Johansen-Berg (2011)). Both methods have distinct advantages and disadvantages that must be considered when interpreting results.

The confirmation of tracts via Klingler’s dissection is perhaps the most reliable evidence of the existence, course, and termination points of white matter fascicles (Agrawal et al., 2011;Klingler & Gloor, 1960;Yendiki et al., 2022), making it a cornerstone of modern neuroanatomy (Jitsuishi et al., 2020;Shinohara et al., 2020;Silva & Andrade, 2016;Vergani, Mahmood, et al., 2014). Although time consuming and dependent on a great deal of technical skill and limited tissue samples (Dziedzic et al., 2021;Judaš et al., 2011;Wysiadecki et al., 2019), it provides specific characterizations of axonal morphology even with small sample sizes.

However, dissection can have difficulty disentangling regions with crossing (David et al., 2019) or intermingled fibres (Takemura et al., 2017). For instance,Maldonado et al. (2012)failed to identify a continuous superior longitudinal fasciculus, instead proposing a discontinuous series of U-fibres stretching from the SFG to the precuneus and collectively appearing as a single fibre bundle. This proposal has been disputed in other work (Janelle et al., 2022). A study byDavid et al. (2019)found tractographic evidence of a supero-anterior fasciculus stretching from the parietal lobe to the orbito-frontal cortex. Attempts to reproduce the bundle in dissection, however, were stymied by the abundance of crossing and overlapping fibre bundles. Finally, the vertical occipital fasciculus has evaded precise identification via dissection (Yeatman et al., 2014) until the last decade (Jitsuishi et al., 2020;Takemura et al., 2016;Yeatman et al., 2013). Thus, failure to find a tract in dissection cannot necessarily be taken as evidence that the tract does not exist, and even consistent identifications may oversimplify the actual anatomy of the individual neurons.

Dannhoff et al. (2024)recently pioneered a new, “inside-out” approach to the dissection of SAFs proceeding from the DWM up toward the cortex. This approach was able to precisely distinguish inter-gyral SAFs from the underlying DWM throughout the brain with greater ease than a traditional, cortex-first approach, and could prove an invaluable tool for future, fine-grained dissection of SAFs.

Unlike dissection, which is generally restricted to small sample sizes from elderly adults, tractography has extremely high throughput and is completely non-invasive, allowing application to healthy and diseased populations across the lifespan. This makes it excellent for exploratory and comparative analyses. However, it also suffers from low specificity (Thomas et al., 2014). Even modern imaging approaches and algorithms have been shown to identify non-existent white matter tracts (Maier-Hein et al., 2017). Thus, novel tracts identified by tractography experiment should be confirmed by dissection. The sensitivity of tractography can also be limited, depending on the methodology. Deterministic algorithms such as Fibre Assignment by Continuous Tracking (FACT), popular choices for exploratory studies due to their relatively high specificity, have inherently lower sensitivity than probabilistic alternatives (Thomas et al., 2014).

Tractography algorithms have particular difficulty resolving crossing fibre populations within a single voxel (J.-D. Tournier et al., 2011), a phenomenon present in as many as 90% of the voxels in a typical dMRI acquisition (Jeurissen et al., 2013;K. G. Schilling, Tax, et al., 2022). More sophisticated diffusion response models such as constrained spherical deconvolution (J. D. Tournier et al., 2007) or the ball-and-stick model (Behrens et al., 2007) perform better than tensor-based tractography, but do not fully eliminate the problem and require more complex acquisitions not available to many clinical studies. This challenge is especially germane to analysis of the SWM. Despite the complex mesh of fibres observed histologically, in dMRI, transverse fibres are by far the most prominent (Cottaar et al., 2018) across the entirety of the GMWMI except for the gyral crown (Fig. 1A). This prevents the accurate tracking of fibres along the GMWMI (Reveley et al., 2015) and leads to a gyral bias (K. Schilling et al., 2018).

In summary, dissection and tractography are complementary approaches, each necessary to map and catalogue the SAFs. Tractography is often used as an exploratory tool and, more recently, has been used to study inter-individual variability in SAFs. Dissection is necessary to validate novel tracts (Yendiki et al., 2022), and provides a more precise account of a tract’s course and termination points. Together, these tools have catalogued local cortical connectivity with great precision, albeit with persistent challenges, as described in the next section.

### 3.3 Association fibres in the SWM

To delineate a white matter tract, it must be extricated from the other tracts sharing its space. The experimenter must prove the tract in question is best defined as a unique unit rather than a mere branch of a larger fascicle. The difficulty of disentangling intermingled fibres using dissection (Vergani, Mahmood, et al., 2014) and the propensity for false positives in tractography studies has hindered the unequivocal identification of some tracts. For instance, the vertical occipital fasciculus, which connects the cuneus to the fusiform gyrus (Jitsuishi et al., 2020), has been a subject of discussion for more than a century, being variously affirmed or dismissed as a part of the inferior longitudinal fasciculus (for a history, seeYeatman et al. (2014)). Only recently has its existence reached consensus, based on its unique histological properties (Yeatman et al., 2014), consistent identification (Takemura et al., 2016;Y. Wu et al., 2016;Yeatman et al., 2014), and a nascent understanding of its role in visual processing (Abdolalizadeh et al., 2022).

Conversely, the full boundaries and extent of a tract are somewhat arbitrary, subject to the constraints of the describing method and the intent of the anatomist. The sledge runner tract, connecting the inferior aspect of the cuneus to the junction of the lingual gyrus and parahippocampal gyrus, was only discovered in the last decade (Vergani, Mahmood, et al., 2014), but has now been repeatedly described by both dissection and tractography experiments (Baydin et al., 2017;Bugain et al., 2021;Koutsarnakis et al., 2019). More recently, however,Beyh et al. (2022)have argued the tract should be considered a subunit of a larger tract called the medial occipital longitudinal tract, connecting both the cuneus and the posterior lingual gyrus to the parahippocampal gyrus, based on a functional understanding of visual processing. Indeed, the anatomy of occipital connections has been tightly linked to visual processing, lending weight to this argument (Movahedian Attar et al., 2020). However, the proposal awaits further consensus.

The FAT, which connects the supplementary motor area to the pars opercularis and pars triangularis (Bozkurt et al., 2017;Catani et al., 2012;Monroy-Sosa et al., 2020;Rojkova et al., 2016;Vergani, Lacerda, et al., 2014), has received prominent attention in the literature due to its important role in language (Catani et al., 2013) and its oblique course across the frontal lobe, which requires special attention in neurosurgery (Monroy-Sosa et al., 2020). As with the vertical occipital fasciculus, there have been recent moves to generalize the FAT as the extended FAT, connecting the entire SFG to the entire inferior frontal gyrus (Catena Baudo et al., 2023;Pascual-Diaz et al., 2020;Varriano et al., 2020). Again, the proposal is made based on functional arguments, specifically the role of the FAT in language (Catani et al., 2013;Catena Baudo et al., 2023).

The MAFs discussed above have received considerable focus. SAFs, on the other hand, have received less attention due to their ubiquity and uncertainty regarding their function. Of these tracts, the most robustly characterized are those connecting the pre-central gyrus to the post-central gyrus under the central sulcus. Although U-fibres line the entire sulcus, three major clusters can be distinguished. The paracentral tract lies in the foot portion of the homunculus; the superior, middle, and inferior hand tracts constitute the hand knob region; and face tracts, sometimes split into superior and inferior subtracts, occupy the ventral aspect of the pre- and post-central gyri in the face portion of the homunculus (Catani et al., 2012;Guevara et al., 2017;Magro et al., 2012;Pron et al., 2021;Rojkova et al., 2016;Román et al., 2017;Thompson et al., 2017;Vergani, Lacerda, et al., 2014). The hand knob region has the highest density of fibres (Magro et al., 2012), perhaps reflecting the higher dexterity of the hand compared with other parts of the homunculus. The explicit functional association with the hand, foot, and mouth portions of the homunculus has been confirmed byPron et al. (2021), who found clusters of increased U-fibre connectivity specifically at cortical sites functionally associated by fMRI with sensorimotor activity in these regions. Additionally, the position of the left hemisphere hand knob was associated with the handedness of the participant. Finally, in patients with autism spectrum disorder (ASD), fractional anisotropy (FA) reductions specifically in the hand knob were linked with reduced dexterity scores (Thompson et al., 2017). Thus, these fibres appear to play a direct role in motor control.

Of the four major cortical lobes, the temporal lobe has received the least attention specific to the SWM. Tracts connecting the insular cortex to the medial aspect of the temporal lobe have been identified (Nachtergaele et al., 2019), andCatani (2022)has reviewed several white matter systems in the temporal lobe, including the temporal longitudinal fasciculus (Maffei et al., 2017), connecting posterior to anterior aspects of the superior temporal gyrus, middle temporal gyrus, and ITG; the fusilum (Epelbaum et al., 2008), connecting posterior to anterior aspects of the fusiform gyrus; and the temporal vertical tract, a system of U-fibres under the superior temporal sulcus (Catani, 2022). To our knowledge, these tracts have yet to be explicitly studied with tractography or dissection.

The ubiquity of SAFs challenges any discrete catalogue or classification, including our own summary inTable 1. Attempts to define bundles of white matter fibres, especially methodologies treating these bundles as isolated streamlines, will tend to ignore the sheet-like character of the SAFs. Some atlasing attempts deal with this by defining large, lobe-sized systems of SAFs, rather than bundles (F. Zhang et al., 2018). This, however, ignores the heterogeneous fibre density observed across the SWM. Some SAF loci, for instance, can be reconstructed in tractography more consistently than others (Pardo et al., 2013).

A recent dissection project byShinohara et al. (2020)proposed an appealing paradigm to handle this tension. They likened the geometry of the gyral folds to the geography of a mountain range. After decortication, they identified gyral junctions (mountain peaks) connected by ridges, together forming a mesh. The spaces in the mesh were filled with classical U-fibres (valleys). Junctions were found at gyral intersections, including 3- and 4-way intersections and small, discontinuous branches. The ridges connecting them were formed by the ascending tracts of inter-gyral U-fibres but contained intra-gyral U-fibres connecting adjacent junctions. SAFs could thus be classified into U-fibres connecting junctions, either intra- or inter-gyral, or inter-gyral U-fibres connecting ridges.

This view of the SWM may provide a more robust, anatomically based organizational paradigm to map SAFs across individuals. Thus far, no other study has attempted to replicate or validate the schema or apply it to SAF atlasing. There is also no study on the functional relevance of their junctions and ridges. Junctions could thus be important epicentres of cortical activity, or inconsequential artefacts of cortical morphology.

SAF density has also been tightly linked to*pli de passages*, small gyri buried within anatomical sulci and thus normally hidden from external view (Mangin et al., 2019).Bodin et al. (2021)have reported the results of a tractography experiment investigating*pli de passages*in the superior temporal sulcus. They found a disproportionately high number of streamlines traversing through*pli de passages*, highlighting their apparent role as high-traffic conduits for white matter connectivity.Pron et al. (2021)also noted that the central white matter cluster of the hand knob travelled through a*pli de passage*in the central sulcus, and that the anatomical location of this central sulcus co-varied with the hand knob according to subject handedness. Thus, a more complete understanding of the cortical folding architecture may further aid the cataloguing of SAFs.

### 3.4 Recent innovations in SWM tractography

Several tractography-related approaches and techniques have been developed in recent years to address challenges specific to SWM tractography, including the impact of gyral bias, the efficient tracking of SAFs by the exclusion of long-range fibres, and the identification of correspondence between highly heterogeneous SAF architectures across subjects. We spend the rest of this section discussing these developments.

#### 3.4.1 Reducing the effect of gyral bias

*Surface Enhanced Tractography*(St-Onge et al., 2018) attempts to diminish the effects of gyral bias by using an analytical prior, rather than the diffusion data, to propagate tracts within the gyral blades. The GMWMI is tightly eroded into a white matter cone within the blade, forming a virtual SWM–DWM interface within the confines of the gyri. Tractography within this interface is conducted as normal. Superficial to the interface, white matter tracts are assumed to evenly fan from the cone to the GMWMI. The two paradigms are merged to form a single tractography. Notably, the approach is agnostic to tractography algorithm, allowing its use as a “plugin” for experiments that may be sensitive to gyral bias. An example application comes fromCole et al. (2021), who employed it to ensure even streamline distribution across the GMWMI in a vertex-wise study of structural connectivity.

*Active Cortex Tractography*(Y. Wu et al., 2021) takes a different approach to limit gyral bias. The method uses a multi-tissue constrained spherical deconvolution approach like that used in*MRtrix*(Jeurissen et al., 2014;J.-D. Tournier et al., 2019). Unlike*MRtrix*, it uses asymmetric fibre orientation distributions (FODs), previously found by the same authors to reduce gyral bias (Y. Wu et al., 2018). It performs streamline tracking using a unique “scouting” approach. Rather than directly applying a streamline propagation algorithm, they defined each streamline step using the interpolation of the next two steps given by the algorithm. This allows streamlines to make tighter turns than the angle cutoff would normally allow, without adversely increasing sensitivity to noise. (In fact, it would in principle reduce noise in long, straight streamline regions, although this was not tested by the authors.) The approach qualitatively increased coverage across the cortical surface, however, to our knowledge its effect on gyral bias has not been extensively quantitatively tested, and it has not been applied to any connectivity study.

#### 3.4.2 Optimizing the delineation of SAFs

*Surface-based Tracking*(Shastin et al., 2022) is an SAF-optimized tractography approach based on*MRtrix*probabilistic anatomically constrained tractography (ACT) (Smith et al., 2012). It uses vertices on the*FreeSurfer*-derived GMWMI mesh (Fischl et al., 1999) as tractography seeds, limits streamline length to 40 mm, and relaxes ACT constraints by allowing tracts to stream in and out of the white matter boundary before reaching a final termination point. Specifically, streamline termination points are calculated as the point on the GMWMI at which the streamline leaves the white matter without returning, rather than the first point at which the streamline leaves the white matter. Compared with traditional tractography approaches, the method tends toward longer, U-shaped streamlines connecting adjacent gyral crowns, thus providing an efficient approach to retrieve the U-fibres classically associated with SAF. On the other hand, it aggravates the gyral bias and compounds the impact of partial volume effects, given the freedom for streamlines to travel in and out of the grey matter. Thus, surface-based tracking is less suited for experiments that sample along the streamlines but may be appropriate for studying changes in streamline geometry and distribution. Thus far, while some studies have performed tractography seeding from the*FreeSurfer*mesh as described byShastin et al. (2022;K. G. Schilling, Archer, Rheault, et al., 2023;K. G. Schilling, Archer, Yeh, et al., 2023), no studies to our knowledge have incorporated their relaxed version of ACT.

Prior to the work ofShastin et al. (2022),Gahm and Shi (2019)published a tractography approach also called*Surface-based Tracking*(Nie et al., 2023). This method was also derived from the*MRtrix*constrained spherical deconvolution approach, but rather than propagating streamlines in 3D space, they first projected adjacent FODs onto the*FreeSurfer*white matter mesh. FOD components radial to the mesh were discarded. Tractography was then performed over this derived, 2D manifold, resulting in a flat tractography reflecting only the diffusion components immediately tangential to the GMWMI. In their most recent report, the same group demonstrated a reduction of the projected FOD correlating with increased severity of tau pathology and clinical disease scores (Nie et al., 2023); however, to our knowledge the technique has not been applied in any other study of the SWM.

#### 3.4.3 Inter-subject correspondence of SWM tracts using atlasing

Comparing MR imaging results across subjects requires the alignment and registration of subject-specific data to a common atlas. Most studies to date achieve this either by segmenting volumetric image data into an atlas of choice, or, for surface-based experiments, by parcellating the cortical surface. A parcellated cortical surface can be used in conjunction with tractography to create a structural connectome. The fibre bundles so delineated will bear some correspondence to white matter tracts formally defined in anatomical studies but are selected solely by their termination points, not by their overall course. Because cortical atlases afford no control over the morphology of the white matter bundles composing each connection, they are not an appropriate tool for the study of white matter fascicles as such.

The past 15 years have seen the rise of white matter atlases designed to address this problem. These atlases are created by running a clustering algorithm over tractography data, typically pooled over several subjects. Streamlines that share similar morphology and endpoints are grouped together into clusters. Clusters that are replicable across subjects are retained for the final atlas. After the desired number of stable clusters is defined, a labelling procedure will be followed, either automatically tagging clusters according to their interactions with cortical regions, or manual naming by a neuroanatomist. Future datasets can then be quickly segmented via clustering with the white matter atlas, allowing reproducible extraction of anatomical tracts.

An in-depth review and comparison of white matter atlas approaches specific to SWM has recently been contributed byGuevara et al. (2020). Here, we will review the developments made since then.

Much recent work has been dedicated to machine learning clustering approaches to improve the speed and accuracy of segmentation.Xue et al. (2023)developed a machine learning paradigm to cluster SWM streamlines according to the O’Donnell Research Group white matter atlas (F. Zhang et al., 2018). While machine learning methods have been used for some time (for review, seeGhazi et al. (2023)), the authors improved on previous methods by first labelling all streamlines as belonging to either the SWM or DWM, and retaining only the SWM streamlines. A more conventional machine learning labelling approach was then applied to the retained fibres. The resulting algorithm was faster than the reference methods, reasonably generalizable across datasets (although datasets similar to the Human Connectome Project dataset: multi-shell, high angular-resolution diffusion imaging (HARDI) data, performed best), and achieved at least a 93% cluster identification rate in healthy adult datasets.

Most clustering work thus far has been done solely on deterministic data, which does not fully reconstruct the entire U-fibre network (Guevara et al., 2020). Guevara’s group has more recently created an SWM atlas with probabilistic data, using multiple clustering and filtering steps to reduce noise and dimensionality (Mendoza et al., 2021;Román et al., 2022;Vázquez et al., 2020). Application of the atlas to new data was optimized with rather aggressive filtering, discarding as many as 50% of the incoming streamlines (Mendoza et al., 2024). The most reproducible filter was a convex hull algorithm (Kai et al., 2022) which discarded fibres with trajectories far removed from the overall shape of the bundle. Importantly, their work showed that segmenting consistent, reproducible bundles improves sensitivity when sampling microstructural measures. Comparing healthy subjects with autistic counterparts, filtered bundles were more likely to have significantly lower FA (Mendoza et al., 2024).

Unfortunately, current algorithms are designed to segment tracts with consistent cross-subject locations and shapes. This works well for long association fibres, the cores of which follow a very consistent course relative to tract volume. SAF location and morphology, on the other hand, vary significantly (Pron et al., 2021), comporting with the highly individualized folding patterns (Wachinger et al., 2015) of gyral architecture (Guevara et al., 2022;Mangin et al., 2019). For instance, the left hemisphere hand knob in the central sulcus and its associated SAFs occupy a more dorsal location in right-handed individuals than in left-handed individuals (Pron et al., 2021;Sun et al., 2012). Neglect of this variability will inherently limit the generalizability of white matter atlases.

Guevara et al. (2022)recently implemented a prototype solution on the SAF bundles in the central sulcus and superior temporal sulcus. Subjects were initially sorted into groups based on sulcal shape and the distribution of SAF bundles crossing the sulcus. Correspondence between bundles was then established, first within subject groups, then across all groups. This stratified approach identified more consistently shaped bundles across subjects than a traditional, whole-group clustering approach.

Nie et al. (2023)developed a clustering technique building on the group’s previous work running tractography on a 2D mesh representation of the SWM. By using the*FreeSurfer*registration technique, which is much more accurate for the cortical surface than traditional volume-based approaches, they were able to project their tractography to a sphere and perform clustering directly on the spherical surface, creating better correspondence between gyri (Y. Li et al., 2023). In their initial report, the method slightly outperformed a more traditional 3D clustering using*QuickBundles*, however, their test was performed on central sulcus U-fibres, which have a fairly stable form across subjects more amenable to volumetric clustering. Their method may hold greater potential for more irregular gyri.

The above approaches have both strengths and weaknesses. Guevara’s method performs well on a single sulcus, but attempting to stratify subjects based on the entire superficial connectome may not be feasible. Compounding variation across the various sulci would prevent the drawing of meaningful equivalence between any two subjects (Wachinger et al., 2015). Individual stratification of each sulcus, followed by the merging of sulcal subtypes to match a particular subject’s brain, may overcome this, assuming sulcal configurations are relatively independent from each other. Li’s approach embraces the fact that SAF morphology is directly linked to GMWMI geometry (Bodin et al., 2021) and incorporates that information elegantly into its clustering algorithm. However, it is limited by its sole use of diffusivity parallel to the cortical surface, eliminating all of the tangential information. We believe the ideal solution will incorporate both the surface information and the 3D tractography information; such an approach has yet to fully materialize.

## 4 SWM in Healthy Development and Disease

### 4.1 SWM development

#### 4.1.1 Pre-natal

**Fig. 2.**
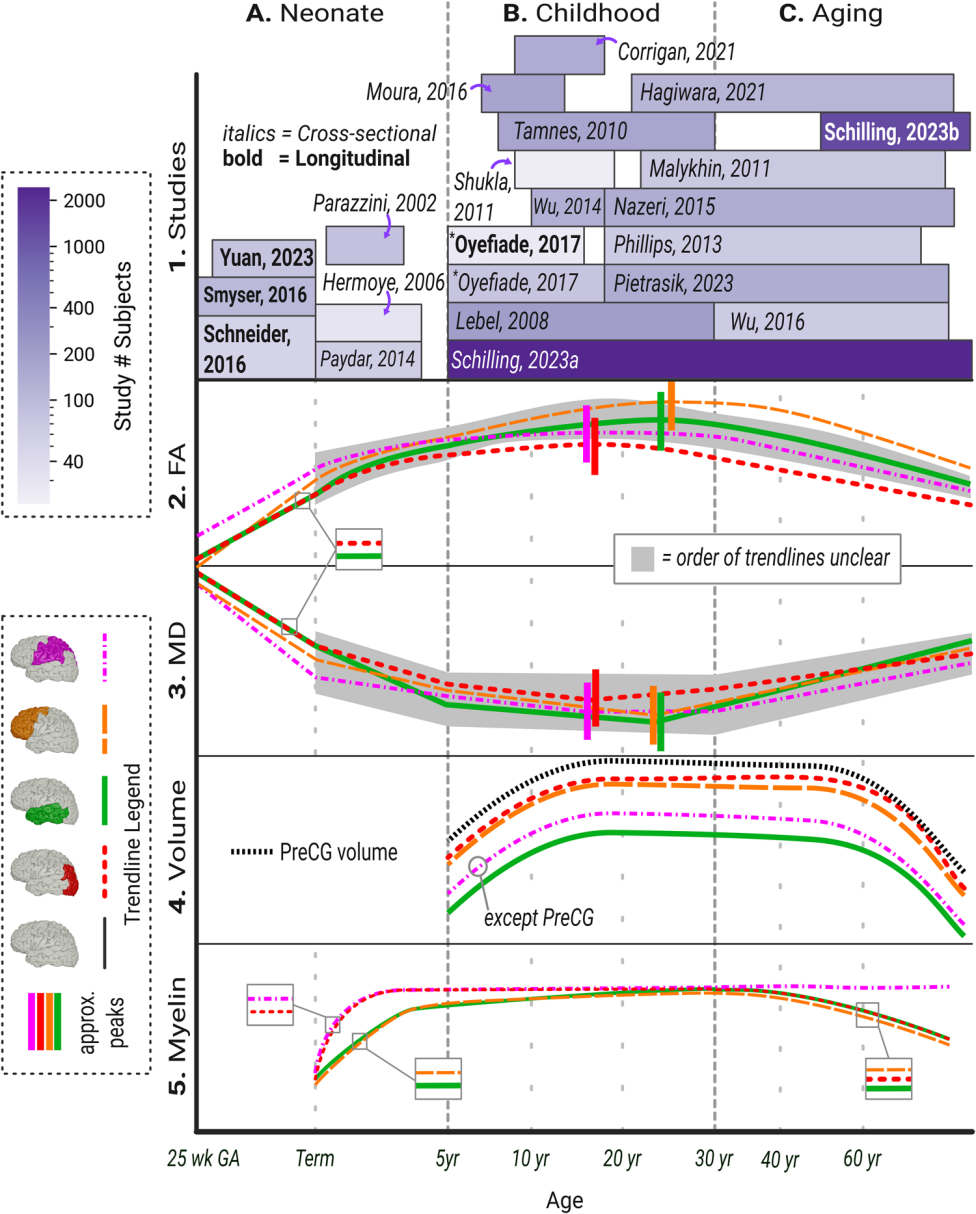
Illustration outlining the general developmental trajectories of various imaging parameters. Column (A): neonates and early childhood (25 weeks GA-5 years). (B): childhood and adolescence (5–30 years). (C): ageing (30–100 years). Row A: Start and end of each box correspond to the age range of the study cohort. Colour corresponds to the number of participants. ((Oyefiade et al., 2018), starred in the figure, have a cross-sectional and longitudinal component; they are listed separately). Studies listed are summarized inTable 2. Rows 2 and 3: FA and MD. Call-out boxes are used to clarify overlapping lines. Row 4: overall superficial white matter volume. In this row only, the purple hatched line should not be interpreted as including the PreCG, which is given its own line. Row 5: overall myelin levels, as determined by varying datatypes, including T2 hyperpolarization in A, magnetic transfer ratio (MTR) and macromolecular proton fraction in B, and MTR in C. All y-axes represent relative, approximate units. All trendlines are approximations from the cited literature. GA = gestational age; PreCG = pre-central gyrus; FA = fractional anisotropy; MD = mean diffusivity.

Neuroimaging results from pre-term babies concur with the developmental picture established by histological studies (an overview of diffusion-weighted imaging methods can be found inBox 1). FA measurements in the SWM are lowest at 25 weeks and increase linearly with time until term (Fig. 2-A.2) (Schneider et al., 2016;Smyser et al., 2016;Yuan et al., 2023). This trajectory aligns with the retreat of the complex microenvironment of the subplate and its replacement with the relatively ordered white matter tracts. The opposite trajectory is observed in the grey matter. FA starts high in mid-gestation and declines toward birth (Ouyang et al., 2019;Schneider et al., 2016;Smyser et al., 2016;Yuan et al., 2023), with some studies reporting a plateau at about 38 weeks (Ouyang et al., 2019;Schneider et al., 2016). These findings likely correspond to the transformation of the radial glia, the dominant diffusive component mid-gestation, into astrocytes.

Box 1 **Interpreting diffusion-weighted imaging in the SWM**

Although many modalities have been used to explore the SWM in health and disease, diffusion-weighted imaging has been especially popular for the rich information it provides and the relative ease of its acquisition. Common to all diffusion methods is the measurement of the diffusion of water in 3D space across one or more spatial scales. Different directions are sampled using orientation-specific encoding gradients, and the scale is determined by the strength and timing of the gradient, summarized mathematically by the b-value. Higher b-values are sensitive to increasingly smaller scales of diffusion. In free space, water will diffuse equally in all directions, but in a restricted microenvironment like the brain, cellular compartments create barriers for diffusion, resulting in a response signal dependent on the orientation and scale of observation. This signal can be used to model the microstructure, inferring what structure would have led to the observed signal (Alexander et al., 2019;Bihan & Iima, 2015;Jones et al., 2013).

Different modelling approaches incorporate varying levels of complexity. The most common approach, DTI, fits a 3D tensor at each voxel, representing the signal as an ovoid shape. From this tensor, various metrics can be computed, including FA, a value between 0 and 1 where 0 corresponds to a completely isotropic (i.e., spherical) diffusion signal and 1 corresponds to a completely anisotropic (i.e., 1-dimensional) signal. FA is high in regions with tightly packed, coherent fibres, notably the corpus callosum and other major white matter tracts. Reduced FA is often interpreted as a reduction in fibre integrity or reduced myelination; however, it is also reduced by increased somatic density, glial density, and by crossing axonal tracts. Because of its heterogeneous composition, the latter likely plays a more determining role in the SWM than the DWM. MD is the mean magnitude of the three eigenvectors, AxD is the magnitude of the longest (in principle lying parallel to the primary axonal population), and RD is the mean magnitude of the two short eigenvectors (lying perpendicular to the primary axonal population) (O’Donnell & Westin, 2011).

DTI assumes that all diffusion follows a Gaussian distribution. This assumption does not hold in complex tissue however, where barriers to diffusion constrain water within a small spatial extent. This results in excess kurtosis: fewer water molecules than expected will diffuse a given distance. In practice, this effect manifests only at b-values higher than those used by DTI (usually b >2000 s/mm^2^). Diffusion kurtosis imaging explicitly measures this phenomenon by sampling both low and high b-values to derive a parameter called mean kurtosis, the magnitude of which is proportionate to the local tissue complexity. In principle, this measure should be higher in the SWM than in the DWM. Although it captures a level of tissue complexity not observable by DTI, it is still quite non-specific and must be interpreted with caution (Steven et al., 2014).

The timing of these developments depends on the region. The time at which FA in the grey matter drops lower than that of the SWM is earliest in primary motor and visual areas (at around 34 weeks) and later in visual association and pre-frontal regions (36–45 weeks) (Smyser et al., 2016). At 28 weeks, FA in the SWM is lowest in the PFC and other association areas, but these regions subsequently have the fastest increases (Fig. 2-A.2) (Yuan et al., 2023). This is consistent with the longer persistence of the subplate in secondary and tertiary association areas (Kostović et al., 2014). Nevertheless, considerable regional heterogeneity remains. The cingulate gyrus, for example, has high FA already at 28 weeks, perhaps reflecting earlier development (Yuan et al., 2023). Converse trends are observed in the grey matter. FA declines fastest in the occipital lobe, frontal lobe, and temporal lobe, possibly somewhat later in the frontal lobe (Ouyang et al., 2019). Cortical FA in the pre- and post-central gyri, cingulate gyrus, and medial frontal lobe starts much lower and declines more slowly, again suggesting earlier development (Yuan et al., 2023).

Mean diffusivity (MD) generally declines in all grey matter and SWM areas throughout the cortex over the third trimester (Fig. 2-A.3) (Schneider et al., 2016;Smyser et al., 2016;Yuan et al., 2023). Mean kurtosis, a measure of microenvironment complexity (Steven et al., 2014), decreases throughout the cortical grey matter (Ouyang et al., 2019). These findings are somewhat harder to interpret but may reflect a reduction in cellular density and complexity in the cortical plate in the weeks before term.

T1 relaxation time declines and the T1/T2 ratio increases throughout the grey matter and SWM at about 35 weeks (Schneider et al., 2016;Yuan et al., 2023). Both measures are often used as a proxy for myelination in the grey matter of adults (Hagiwara et al., 2018), but in the white matter, they have almost no correlation with myelin water fraction (MWF), a more sensitive metric for myelin quantification (Arshad et al., 2017;Uddin et al., 2018). To our knowledge, no definitive histological corollary of T1/T2 signal in the complex pre-term microenvironment has been found, preventing decisive conclusions from these data.

Note that the developmental trajectory of pre-term babies may differ from normally developing children. Indeed, previous work has found extensive reductions in FA throughout the white matter in pre-term compared with full-term babies of equivalent age (Anjari et al., 2007). Difficulties in registration and controlling foetal movement currently make*in utero*scanning very difficult, but should these difficulties be resolved, such an approach may give a more accurate picture of normal pre-natal development (Stout et al., 2021).

#### 4.1.2 Childhood

The most substantial changes in the aforementioned parameters occur during the pre-natal period and first few years of life (Lebel & Deoni, 2018). U-fibres undetectable on diffusion imaging at 3 months post-birth have near mature FA levels well within the first year (Hermoye et al., 2006). Across the whole white matter, FA and mean kurtosis reach stable plateaus between 1 and 3 years (Fig. 2-A.2, A.3) (Paydar et al., 2014).

Nevertheless, changes continue to occur throughout the brain over the course of childhood development and into adolescence, albeit at a slower rate (Lebel et al., 2008). FA linearly increases in SWM across the cortex, while MD and axial diffusivity (AxD) decrease (Fig. 2-B.2, B.3) (Oyefiade et al., 2018;Shukla et al., 2011;Tamnes et al., 2010;M. Wu et al., 2014). Both FA and MD eventually reach a stable developmental plateau lasting a few decades, before declining in old age (K. G. Schilling, Archer, Rheault, et al., 2023). This plateau arrives sooner (between 10 and 20 years of age) for primary motor and visual areas, and later (>20 years) for the frontal lobe, temporal lobe, cingulate gyrus, and other association areas (Tamnes et al., 2010).

SWM thickness increases by about 0.5% per year until about age 20 years (K. G. Schilling, Archer, Rheault, et al., 2023), such that in most regions, the total SWM volume increases by 10–20% between the ages 8 and 30 years (Fig. 2-B.4) (Tamnes et al., 2010). This growth proceeds slower than the overall expansion of the white matter, so that SWM volume relative to the total white matter decreases from 5 to 15 years. (Ouyang et al., 2016;K. G. Schilling, Archer, Rheault, et al., 2023). Throughout, SWM thickness is highest in the pre-central gyrus, followed by the frontal lobe, post-central gyrus, and occipital lobe, than the parietal lobe and temporal lobe (K. G. Schilling, Archer, Rheault, et al., 2023).

The changes observed beyond early childhood are quite subtle. The annualized rate of FA change across adolescence, for instance, generally ranges between 0.5% and 1%, and R^2^values are usually below 0.25 (Tamnes et al., 2010;M. Wu et al., 2014), leaving a significant amount of individual variability. Thus, these changes can currently only be observed over decade-long time periods. This is highlighted by a recent study byOyefiade et al. (2018), which found a significant increase in FA in all lobes except the occipital lobe using a cross-sectional sample of 5- to 18-year-old participants but almost no significant differences in a longitudinal analysis of equivalently aged subjects scanned over a 5-year timeframe.

#### 4.1.3 Myelination

Although its precursors are visible as early as 20 weeks post-conception, the slow progression of myelination continues throughout a child’s first few years, possibly extending beyond adolescence. As shown by histological studies, myelination proceeds from caudal to rostral regions, from deep to superficial, in sensory pathways before motor, and in projection before association fibres (Kinney et al., 1988;Martin et al., 1988). Thus, SWM, especially in the frontal lobe, is the last of the white matter to myelinate. While most myelination is complete by about 18 months, the SWM and peritrogonal areas, known as the terminal regions, have a slower course of development. T2 hyperintensity, associated with a lack of myelin, only disappears from the peritrogonal areas and parietal SWM by 20 months post-natal. Hyperintensities linger in the frontal lobe and temporal lobe until around 40 months (Fig. 2-A.5) (Maricich et al., 2007;Parazzini et al., 2002).

The long-term course of SWM myelination remains relatively uncharacterized. Studies frequently claim the myelination process continues until the third or fourth decade of life, an idea originating from an early histological study that observed unmyelinated association fibres in subjects so aged (Yakovlev, 1967). As mentioned above, certain regions of the SWM do follow a longer developmental trajectory (Dubois et al., 2014), and in general, the central, deeper portions of white matter tracts have greater age-related changes in early childhood than the cortical termination points (Geng et al., 2012). Histological observations in adolescents confirm the SWM remains relatively less myelinated than the DWM within the first two decades of life. (Sarnat et al., 2018). However, it is not clear whether this lower myelination reflects a longer developmental timeframe or characterizes a normal, mature SWM. Maturational delay certainly does not appear to be a universal feature of the SWM. For instance, a Tract-Based Spatial Statistics (TBSS) analysis of magnetization transfer ratio (MTR) in children of ages 7–14 years failed to find any effect of age, either in deep or superficial regions (Moura et al., 2016). In likely the most specific brain-wide study published to date,Corrigan et al. (2021)characterized a well-validated, myelin-sensitive parameter, macromolecular proton fraction (Kisel et al., 2022;Yarnykh, 2002), across the grey matter and SWM. They observed a spatially widespread, positive effect of age on myelin levels throughout the grey matter in participants aged 9–17 years. In the white matter, however, significant effects were limited to the pre- and post-central gyri, orbito-frontal cortex, and right superior temporal gyrus. At a lobe-wide scale, only the temporal lobe was significantly affected. No effect was found in the DWM. Thus, at least some portions of the SWM appear to undergo ongoing myelination throughout adolescence, but the scope of such processes may be limited. Further research is needed to both validate these findings and determine when myelination finally concludes (Fig. 2-B.5).

Furthermore, recent advances in iron-sensitive imaging suggest that reduced myelin may be a defining characteristic of the SWM. A study byKirilina et al. (2020)used histology and high-resolution, quantitative MR imaging (measuring$R_{2}$,$R_{2}^{*}$(Bagnato et al., 2011;Fukunaga et al., 2010), andχ) to demonstrate a prominent band of iron from 0.5 to 2 mm thick immediately beneath the GMWMI in the post-mortem brain of an elderly adult (Fig. 1-C). Outside of this band, myelin signal in the white matter was at a stable, DWM level. Within the band, it linearly dropped to grey matter levels. This finding was recently reproduced in the middle frontal sulcus and pre-central sulcus of young adults 20–30 years old byLee et al. (2023). They used a novel technique to separate the iron and myelin contributions to quantitative susceptibility mapping (QSM) (χ-separation) (Shin et al., 2021), corroborating the method with MWF. They observed the same iron and myelin patterns asKirilina et al. (2020). Thus, given the reduced SWM myelin levels observed even in an elderly adult (Kirilina et al., 2020), reduced myelin signal near the GMWMI may be a persistent feature throughout life. However, no study of a myelin-sensitive parameter, such as MTR or MWF, has yet reported a specific comparison between SWM and DWM levels across the lifespan.

The cause of persistently reduced SWM myelin throughout life, assuming the existence of the phenomenon, is open to speculation. The SWM forms a transition zone between the DWM and the grey matter. WMIN density increases closer to the GMWMI, and the dominant diffusion signal, oriented tangentially to the GMWMI in the SWM, gives way to the radially oriented signal in the grey matter. Thus, the linear drop in myelin signal may simply reflect the SWM becoming more “grey matter-like” as it nears the GMWMI. Increased cell density restricting the spatial extent of myelin (causing partial volume-like effects on imaging), increased density of unmyelinated interneurons (Smiley et al., 1998), or reduced myelin levels across some or all axons could play roles. More speculatively, high iron levels colocalized with increased oligodendrocyte density (Kirilina et al., 2020) provide the machinery for ongoing myelination (J. R. Connor & Menzies, 1996). This could be the product of myelin flux as connections are continually formed and pruned.

#### 4.1.4 Cortical folding

The relative consistency of the cortical folding pattern and the differences in SWM between gyral blades and under sulcal fundi has inspired hypotheses relating SAFs to the formation of gyral folds. The tension-based morphogenesis hypothesis, among the most prominent, suggests that axonal tension arising from strongly connected areas directly contributes to the stereotyped folds observed in humans (Essen, 1997). Cortical folding is beyond the scope of this article but has been reviewed elsewhere (Llinares-Benadero & Borrell, 2019;Mangin et al., 2019).

### 4.2 SWM and ageing

While diffusivity metrics across the SWM remain relatively consistent post-adolescence, long-term ageing is associated with the decline of FA and the increase of MD and AxD (Fig. 2-C.2, C.3) (Malykhin et al., 2011;Nazeri et al., 2015;Phillips et al., 2013;Pietrasik et al., 2023;K. G. Schilling, Archer, Yeh, et al., 2023). The robustness of this finding has been very recently confirmed in a 1293-subject longitudinal study byK. G. Schilling, Archer, Yeh, et al. (2023), with significant results in every SWM fibre bundle analyzed. As in earlier development, the annualized change is modest, with FA declining at about 0.25% per year. Additionally, myelin-sensitive signals, including MTR and myelin volume fraction, decline in most regions (Fig. 2-C.5) (Hagiwara et al., 2018;M. Wu et al., 2016). Regions with earlier myelin maturation, such as the superficial pre- and post-central gyri, maintain robust myelination longer than other gyri (Kakeda et al., 2016).

Less certain is the age at which these changes begin. All current studies concur they start no later than the age of 50 years in healthy adults (Malykhin et al., 2011;Nazeri et al., 2015;Phillips et al., 2013;Pietrasik et al., 2023;K. G. Schilling, Archer, Rheault, et al., 2023;K. G. Schilling, Archer, Yeh, et al., 2023). However, amongst studies of individuals younger than 50 years, some report changes beginning at age 40–50 years (Malykhin et al., 2011;Pietrasik et al., 2023;K. G. Schilling, Archer, Rheault, et al., 2023;M. Wu et al., 2016), while others report changes as early as 30 years (Nazeri et al., 2015;Phillips et al., 2013). Interestingly, the studies endorsing later onset all use a tractography approach to define the SWM, while the early onset studies use voxel-wise, atlas-based methods such as TBSS. One possible explanation for the discrepancy may be that tractography-based approaches sample a larger, deeper region of white matter, given the lack of absolute volume constraint. This is decidedly the case inMalykhin et al. (2011)andPietrasik et al. (2023), both of which analyze very large white matter bundles, some of which pass through the arcuate fasciculus and corpus callosum. These larger ROIs bias the results toward the time course of DWM ageing, the onset of which has been reported at 40 (Beck et al., 2021) to 50 (Sexton et al., 2014) years of age. Furthermore, there is evidence suggesting that the last white matter fascicles to mature are the first to decline (Bender et al., 2016). Thus, studies defining SWM to a narrower volume may find earlier changes than those sampling from deeper white matter. This hypothesis must be confirmed by future experiments.

Changes in the SWM have been associated with cognitive decline in otherwise healthy adults.Nazeri et al. (2015)reported a correlation between FA in the parieto-occipital areas and visuomotor attention. FA in the pre- and post-central gyri was correlated with fine motor performance. Given the cross-sectional dataset used, these results inform us about the effect of diffusion tensor imaging (DTI) metrics on cognitive performance without respect to age. To our knowledge, no one has used longitudinal data to study the effects of individual age-related DTI changes on cognitive decline.

### 4.3 SWM and disease

The collateralized blood supply of the SWM and the relatively late maturation of its microstructure give it a unique profile of vulnerability to disease compared with the DWM and cortex. For example, the SWM is typically spared from small vessel disease otherwise common in the white matter. Certain genetic disorders affecting the metabolism and production of myelin, including metachromatic leukodystrophy and X-linked adrenoleukodystropy, also spare the SWM until later in the disease course, due to its slower rate of myelin metabolism. Such diseases have been reviewed elsewhere (Riley et al., 2018). Here, we will focus on diseases that have been studied using neuroimaging, especially dMRI studies that have looked particularly at the SWM (Ouyang et al., 2017).

#### 4.3.1 Schizophrenia

Schizophrenia is a chronic disorder associated with positive (delusions, hallucinations, thought and language disorder, etc.) and negative (anhedonia, alogia, etc.) symptoms (Keshavan et al., 2008;Tandon et al., 2009). A previous mega-analysis has reported a significant, substantial reduction of FA in the DWM, especially affecting callosal and long association fibre systems (Kelly et al., 2018). Far fewer studies have specifically investigated the SWM, but their collective findings suggest a reduction in superficial FA throughout the frontal lobe (Ji et al., 2019;Joo et al., 2023;Kai et al., 2023;Nazeri et al., 2013). A few studies additionally found reduced FA in the occipital lobe (Joo et al., 2023;Nazeri et al., 2013;Phillips et al., 2011), parietal lobe (Joo et al., 2023), and precuneus (Ji et al., 2019;Nazeri et al., 2013). Methodologies vary; older studies used TBSS (Nazeri et al., 2013) and mesh projection (Phillips et al., 2011), while newer studies (Ji et al., 2019;Kai et al., 2023) have taken advantage of SWM bundle atlases (Guevara et al., 2017;F. Zhang et al., 2018).

Kai et al. (2023)reported relatively little difference between early-stage schizophrenia patients and controls; however, their study was performed on a first-episode, mostly untreated sample, indicating a difference between this population and their chronic counterparts. Supporting this notion,Ji et al. (2019)found lower FA in certain SWM fibre bundles in patients with more olanzapine equivalents. Because the study was cross-sectional, it cannot prove that medication or disease exposure directly lowers FA. Pre-existing low FA may alternatively increase susceptibility toward longer disease duration or reduced treatment response. However, a study byHalene et al. (2016)in macaques reported a direct effect of clozapine exposure on increased WMIN density, a phenomenon which would, in principle, decrease SWM FA. Nevertheless, no replication of this finding has been made in humans, and no longitudinal studies of SWM diffusion metrics in schizophrenia have been performed, rendering these hypotheses speculative.

The lower SWM FA reported in patients may reflect somatic changes rather than changes in myelination or axonal organization. Compared with controls, SWM in the dorsolateral PFC of patients has increased WMIN density (Duchatel et al., 2019;Eastwood & Harrison, 2003,^2005^;Kirkpatrick et al., 2003;Yang et al., 2011). Increased WMIN density can also be seen in the superior temporal gyrus (Eastwood & Harrison, 2003), the cingulate gyrus (C. M. Connor et al., 2009), and the orbito-frontal cortex (Joshi et al., 2012).

This increase in WMIN density is accompanied by the decline of certain grey matter neural populations, including somatostatin+ (Yang et al., 2011), Dlx1, and GAD76 (Joshi et al., 2012) neurons, all of which are GABAergic inhibitory neurons. Such mirrored effects in the two layers may reflect failed migration of interneurons from white to grey matter (Carrel et al., 2015;Tee & Mackay-Sim, 2021).

Oligodendrocyte density is also increased in the SWM of schizophrenia patients (Bernstein et al., 2012,^2016^). This elevation predicts an increase in SWM iron concentration: much of the SWM iron signal colocalizes with oligodendrocytes (Kirilina et al., 2020), iron is an important cofactor in myelin synthesis, and oligodendrocytes express a protein profile optimized for iron acquisition and utilization (J. R. Connor & Menzies, 1996). Thus, MR imaging methods sensitive to iron levels may be a productive, non-invasive means of future investigation. Histological examination has found a general increase in iron in the PFC grey matter (Lotan et al., 2023), and a few studies have used varying iron-sensitive signals (QSM and an inverse normalized T2) to study schizophrenia, finding increased signal in the putamen (Ravanfar et al., 2022), thalamus (Sonnenschein et al., 2022;Xu et al., 2021), substantia nigra, and red nucleus (Xu et al., 2021). No studies, to our knowledge, have explored iron levels specifically in the SWM. As mentioned above in our discussion of myelination,Shin et al. (2021)have recently developed a method calledχ-separation, which distinguishes the QSM contributions from iron and myelin for independent quantification. The technique was specifically applied to analysis of the SWM (Lee et al., 2023), and should be useful for application in disease studies.

#### 4.3.2 Dementia

Studies of SWM in Alzheimer’s disease (AD) have consistently reported reduced FA and increased MD, radial diffusivity (RD), and AxD throughout the brain, especially in the temporal lobe (Bigham et al., 2020;Contarino et al., 2022;Phillips, Joshi, Piras, et al., 2016;Reginold et al., 2016), although various studies have also implicated the PFC (Phillips, Joshi, Piras, et al., 2016), limbic cortex, insular cortex (Bigham et al., 2020), and parietal lobe (Contarino et al., 2022;Veale et al., 2021). A study using the neurite orientation dispersion and density imaging (NODDI) model (H. Zhang et al., 2012) found reduced neurite density index (NDI) in the parietal lobe and parahippocampal gyrus, reflecting an increased proportion of signal attributed to non-neurite space. It also reported increased orientation dispersion index (ODI) in the parahippocampal gyrus and fusiform gyrus, reflecting a higher distribution of neurite angles (Veale et al., 2021). MTR, a proxy for myelin, is reduced compared with healthy, age-matched controls throughout the brain (Fornari et al., 2012). Additionally, the regional covariance of MTR levels, especially among long-range connections, is reduced, meaning regions geometrically distant from each other tend to have different MTR levels in AD patients (Carmeli et al., 2014).

The diffusivity results align with previously identified histological changes. Nitrinergic cells in the SWM are relatively spared by AD, showing no decline in density and no association with tau-reactive neurofibrillary tangles. They do, however, present disrupted morphology and reduced fibre density (Kowall & Beal, 1988;Norris et al., 1996;Tao et al., 1999), possibly contributing to increased ODI and decreased NDI.

The SWM appears to be more specifically involved in fronto-temporal lobar degeneration (FTLD), specifically the FTLD-TDP subtype, often associated with mutations in the progranulin gene (Baker et al., 2006). Compared with AD, much of the FTLD pathology is subcortical, with extensive microglial activation (Lant et al., 2014;Sakae et al., 2019;Taipa et al., 2017) and histologically observable pathology localized to the white matter (Giannini et al., 2021). Pathological findings notably include thread-like processes that appear to arise from oligodendrocytes (Giannini et al., 2021;Hiji et al., 2008;Mackenzie & Neumann, 2020;Neumann et al., 2007). The pathophysiological significance of these findings is not currently understood. Imaging studies have concurringly observed T2 hyperintensities and T1 hypointensities in the SWM of the frontal lobe (Caroppo et al., 2014) and temporal lobe (Mori et al., 2007). Interestingly, both the imaging results (Domínguez-Vivero et al., 2020;Mori et al., 2007) and the oligodendrocyte pathology (Giannini et al., 2021,^2023^;Hiji et al., 2008) are specific to FTLD-TDP (as opposed to the tau subtype of FTLD and other motor disorders such as amyotrophic lateral sclerosis). This does not prove that the changes on imaging are caused specifically by oligodendrocyte pathology, but the disorder may prove an interesting model for relating imaging to histology.

#### 4.3.3 Autism spectrum disorder

ASD has been generally linked to hyperconnectivity locally, but hypoconnectivity globally (Ouyang et al., 2017). For example, in a TBSS analysis, long skeletal tracts situated in the DWM generally had lower FA, but amongst the shorter tracts in the SWM, only the frontal lobe was affected (Shukla et al., 2011). A histological study, in turn, reported a reduction in long-range, large-diameter neurons in the DWM below the anterior corpus callosum, with a correspondingly increased density of small diameter, short-range fibres in the SWM explainable by increased fibre branching. No significant results were found in the dorsolateral PFC however (Zikopoulos & Barbas, 2010).

Recent studies have linked diffusion parameters in the SWM with task performance and symptom scores.d’Albis et al. (2018)found reduced FA in ASD patients in a structural component incorporating SAFs from the temporal lobe, parietal lobe, and frontal lobe. Lower FA in this component correlated with decreased performance in social awareness, pragmatic skills, and empathy evaluation tests. In an additional component localized to the supplementary motor area and insular cortex, lower FA correlated with reduced language structure and social awareness.Thompson et al. (2017)found a positive correlation between FA in the hand knob fibres and performance in a fine-motor task.Bletsch et al. (2021)associated disease severity with lower FA in the right PFC and temporal lobe and the left fusiform gyrus, inferior temporal gyrus, and orbito-frontal cortex.

#### 4.3.4 Epilepsy

In temporal lobe epilepsy patients, reduced FA and increased MD have been found in the temporo-parietal connection, orbito-frontal cortex, cingulate gyrus, and medial temporal lobe ipsilateral to the epileptic lesion. Contralateral effects were scattered across the medial aspect of the hemisphere, with some lateral FL involvement (Liu et al., 2016;Urquia-Osorio et al., 2022). This reduction in FA has been linked to a decrease in neurite density using the NODDI model and correlated with disease duration (Winston et al., 2020). SWM involvement has also been reported in focal cortical dysplasia, although damage is more restricted to the site of the dysplasia (Urquia-Osorio et al., 2022). Histologically, an increased number of neurons, especially heterotopic neuronal synaptic plexi, can be found in the SWM in the disease, with evidence of increased metabolism. These cells are thought to participate in epileptic circuits (Sarnat et al., 2018). In Rolandic epilepsy, increased MD, RD, AD, and, contrary to the normal pattern, FA were found specifically in the pre- and post-central gyri (Ostrowski et al., 2019). FA furthermore negatively correlated with fine motor skills, opposite the association observed in autism (Thompson et al., 2017).

#### 4.3.5 Other diseases

SWM has been cursorily studied in a number of other diseases. While it is not our intention to provide a complete documentation of these diseases and the potential impacts of SWM involvement, we summarily note previous investigations in Huntington’s disease (Phillips, Joshi, Squitieri, et al., 2016), anti-NMDA receptor encephalitis (Phillips et al., 2018), Parkinson’s disease (Y. Zhang et al., 2022), multiple sclerosis (Buyukturkoglu et al., 2022;Komnenić et al., 2024), Tourette syndrome (Wen et al., 2016), bipolar disease (S. Zhang et al., 2018), traumatic brain injury (Stojanovski et al., 2019), cerebral small vessel disease (S. Wang et al., 2022), multiple system atrophy (Del Campo et al., 2021), and various other developmental and metabolic disorders (Riley et al., 2018). In all cases mentioned above, the authors report reduced FA and a corresponding increased MD, AxD, and RD, the distribution of which depends on the disease. In general, however, the frontal lobe and temporal lobe are very often involved.

## 5 Challenges for Interpretation

### 5.1 Segmenting the superficial and deep white matter

Distinguishing the SWM from the DWM, both in histological studies and neuroimaging, remains a persistent problem. Unlike the GMWMI, easily identified on both T1- and T2-weighted MR images and on histological sections, no sharp delineation exists between the SWM and DWM, and no consistent rule has been adopted to segment the two regions (Meyer et al., 1992;Sedmak & Judaš, 2019). This creates a special challenge for dMRI experiments, often acquired with the rather low 2 mm isotropic resolution and, therefore, vulnerable to partial volume effects. Voxels spanning the SWM–DWM boundary contain mixed signal from both tissue layers, but if this boundary cannot be reliably identified, identification and correction of such voxels become impossible.

Many studies use voxel-based ROIs to define the SWM. A popular segmentation created byOishi et al. (2008)selects mainly the intra-gyral space as the SWM compartment. Others define a layer of fixed width extending from the GMWMI.Sedmak and Judaš (2019)recently proposed a depth of 3 mm, targeting a layer comprising only definitive remnants of the subplate. Other studies have performed such depth sampling up to 5 mm deep (M. Wu et al., 2016). All of these definitions, however, are somewhat arbitrary, ignoring variation across subjects, across the lifespan, and across brain regions. Moreover, they all likely overestimate the thickness of the layer. Recent dissection work byDannhoff et al. (2024)measured SAF thickness underneath the superior temporal sulcus as ranging from 0.5 to 3 mm. Accurate segmentation on imaging would thus require a minimum 0.5 mm isotropic resolution, not currently routinely collected for either anatomical or diffusion protocols.

Tractographic approaches analyze the voxels traversed by tracts of interest. SAFs are selected either using inclusion and exclusion ROIs, or using an SWM atlas (Guevara et al., 2020). The measured SWM thickness depends on the number of voxels occupied by SAF streamlines which, in turn, depends on the image resolution. For studies with the common 2 mm isotropic resolution, this will mean a thickness of 4–8 mm, depending on the region. As mentioned above, this will likely overestimate the actual thickness of the SAF bundles. To our knowledge, the exact relationship between SAF bundle thickness and image resolution has not yet been characterized, so it is not clear whether imaging results will reflect histological ground truth at sufficiently high resolution. If imaging persistently overestimates SAF thickness, perhaps an inevitable consequence of partial volume effects, a corrective factor could potentially be applied, but this would require a normative SAF thickness atlas across the entire brain.

Histological studies have used WMIN cell count as a marker, given the rarity of WMINs in the DWM. Because cell density decreases on a gradient, however, the definition remains rather arbitrary, resulting in wide range of SWM cell counts (Eastwood & Harrison, 2005;García-Marín et al., 2010;Sedmak & Judaš, 2019).

The iron-sensitive imaging results ofKirilina et al. (2020)andLee et al. (2023)suggest a potential quantitative definition of the SWM, based on the hyperintense band of iron deep to the GMWMI and the colocalized drop in myelin signal. This band ranged from 0.5 to 2 mm thick depending on the region and appeared to correspond to an expanded population of oligodendrocytes (Kirilina et al., 2020). It also concurs with the SAF thickness reported inDannhoff et al. (2024). Acquisition of this signal, however, requires a QSM sequence neither routinely collected in imaging paradigms nor commonly available in open datasets.

As the distinction between SWM and DWM inherently exists on a gradient, no single, cross-modality definition will likely ever exist. Unfortunately, however, the diverse segmentation methods used across developmental and disease studies result in dramatically different SWM definitions, varying by volume, thickness, and, in the case of TBSS and tractography studies, sampling weight. Such inconsistent definitions challenge the construct validity of these experiments and limit meaningful comparison between them. Future reliance on SWM atlases will help rectify this, as the SWM definition will be directly based on the presence of SAFs, one of the cardinal features of SWM. Finally, true segmentation of the SWM in diffusion scans will require images of 2–4-fold higher resolution than typically obtained, likely not technically feasible for most studies at this time. Therefore, careful reporting and justification of segmentation method will remain important to ensure replicability and cross-study comparability.

### 5.2 Interpretation of diffusion parameters

The complexity of the tissue in the SWM amplifies the challenge of interpreting basic diffusion parameters such as FA. Indeed, the predominance of reduced FA and increased MD, RD, and AxD across the diverse diseases previously discussed highlights the sheer non-specificity of these markers. Reduced FA in the SWM does not even consistently mark the presence of illness. We previously referred to the study of children with Rolandic epilepsy, where higher FA in the pre- and post-central gyri was associated with worse motor performance (Ostrowski et al., 2019). We can also highlight a study which found reduced FA in the SWM of accomplished gymnasts, compared with non-athletically trained controls (Deng et al., 2018).

This non-specificity may be attributed to the large variety of cellular and molecular factors that influence diffusivity. Many authors associate reduced FA with disruption to white matter integrity, but although this simplistic explanation has some empirical basis in the DWM (Holleran et al., 2017), its assumptions do not hold in the SWM. Interpretations of diffusivity changes must further consider the greatly elevated density of WMINs relative to the DWM, the greater population of oligodendrocytes, and the complex mesh of neural fibres running both tangentially and radially to the GMWMI (Yao et al., 2023). For example, as previously discussed, WMINs of the SWM are affected in schizophrenia patients, likely influencing the FA in the region.

Such interpretive challenges can be mitigated by the use of more specific imaging parameters. Robust markers of myelination, such as MTR and macromolecular proton fraction (Kisel et al., 2022), have been under-used in the SWM disease literature. Recent developments in iron-sensitive imaging may also prove useful, especially if its previously observed colocalization with oligodendrocytes proves replicable. Some studies mentioned have used the NODDI model (H. Zhang et al., 2012) of diffusion (Veale et al., 2021;Winston et al., 2020). This paradigm segments the diffusion signal into neurite and extra-neurite compartments, giving more specific insight into the composition of the tissue. The more recently developed soma and neurite density imaging (SANDI) model (Palombo et al., 2020), as yet unused in the SWM literature, further splits the extra-neurite space into an intra-soma space and an extra-cellular space. Given the high cell density in the SWM, this approach may be especially well suited for future study, however, its derivation requires an extensive, specialized MR sequence with a max b-value of 10,000 s/mm^s^. Finally,J. He and Wang (2024)have proposed a diffusion model incorporating water exchange both across cell membranes and between soma and axons, specifically to account for the unique mixture of neurons and neurites found in the SWM. Their current results appear to accurately delineate the SWM, although this remains to be validated. It has the further advantage over SANDI of requiring fewer, lower b-values, and fewer directions, which would allow a shorter scan time. Although the technology for many of these models may currently be beyond the means of many clinical studies, they offer a promising, more specific look into SWM microstructure moving forward.

### 5.3 Imaging of specific histological characteristics

In general, imaging parameters and particular histological properties do not have any direct correspondence (Lerch et al., 2017) outside of highly specialized acquisitions or very particular disease states. The closest examples discussed in this review include the various myelin-sensitive and iron-sensitive imaging methods. It is, therefore, critical to avoid over-interpretation of imaging results in the absence of ground-truth data. Nevertheless, the general sensitivities of various MR acquisitions to tissue characteristics can and should guide the design of imaging paradigms. InTable 3, we present a non-exhaustive summary of the sensitivities of some mainstream structural MR modalities. Numerous quantitative imaging types are now available, and their delineation with a full discussion of their strengths and weaknesses is well beyond the scope of this review.

**Table 2.** Summary of literature on superficial white matter development.

Reference	Age range	Participants	Design	Modalities
( M. Wu et al., 2014 )	10–18	133	Cross-sectional	FA, MD, AD, RD
( Tamnes et al., 2010 )	8–30	168	Cross-sectional	FA, MD, SWM Volume
( K. G. Schilling, Archer, Yeh, et al., 2023 )	5–100	2421	Cross-sectional	SWM Volume
( Ouyang et al., 2016 )	2–25	21	Cross-sectional	# Short Streamlines / # Total Streamlines
( Oyefiade et al., 2018 )	5–18	78	Cross-sectional	FA, MD, AD
( Oyefiade et al., 2018 )	5–17	26	Longitudinal	FA, MD, AD
( Shukla et al., 2011 )	9–19	24 *	Cross-sectional	FA
( Yuan et al., 2023 )	24GA–Term	78	Longitudinal	FA, MD, T1/T2
( Smyser et al., 2016 )	25GA–Term	105	Longitudinal	FA, MD
( Schneider et al., 2016 )	25GA–Term	51	Longitudinal	T1, FA, ADC
( Parazzini et al., 2002 )	20–40 mn	85	Cross-sectional	T2
( Malykhin et al., 2011 )	22–84	69	Cross-sectional	WM Volume, FA, MD, AD, RD
( Phillips et al., 2013 )	18–74	65	Cross-sectional	FA, AD, RD
( Pietrasik et al., 2023 )	18–85	140	Cross-sectional	FA, MD, AD, RD, WM Volume
( K. G. Schilling, Archer, Rheault, et al., 2023 )	50–98	1293	Longitudinal	FA, MD, AD, RD, SWM Volume
( M. Wu et al., 2016 )	30–85	66	Cross-sectional	MTR
( Paydar et al., 2014 )	0–4	59	Cross-sectional	FA, MK
( Hermoye et al., 2006 )	0–4	30	Cross-sectional	FA, ADC, nb0
( Hagiwara et al., 2021 )	21–86	114	Cross-sectional	T1, T2, PD, MVF
( Lebel et al., 2008 )	5–30	202	Cross-sectional	FA, MD
( Moura et al., 2016 )	7–14	176	Cross-sectional	FA, MD, MTR
( Nazeri et al., 2015 )	18–86	141	Cross-sectional	FA, MD, RD
( Corrigan et al., 2021 )	9–17	146	Cross-sectional	MPF

* Excludes disease arm of study.

GA = gestational age; FA = fractional anisotropy; MD = median diffusivity; AD = axial diffusivity; RD = radial diffusivity; ADC = apparent diffusion coefficient; MVF = myelin volume fraction; MTR = magnetic transfer ratio; PD = proton density; SWM = superficial white matter; MK = mean kurtosis; MPF = macromolecular proton fraction.

**Table 3.** Structural MR acquisitions with an overview of histological sensitivities.

Parameter	Acquisition	Sensitivity	Strengths	Weaknesses
T1, T2, T1/T2	T1 w and T2 w	Myelin ( Glasser & Van Essen, 2011 )	Routinely available, easily calculated.	Highly non-specific: Unsuitable for quantitative use in WM ( Arshad et al., 2017 ; Hagiwara et al., 2018 ; Parent et al., 2023 ; Uddin et al., 2018 ).
R1 (1/qT1)	Quantitative T1 mapping (such as MP2RAGE)	Myelin ( Lutti et al., 2014 ), iron, tissue hydration, protein content ( Hagiwara et al., 2018 ; Hertanu et al., 2022 )	Does not require a special scan for derivation. Better specificity to myelin than regular T1, T2 parameters ( Parent et al., 2023 ).	Not wholly specific to myelin.
MTw parameters (MTR, MPF ( Kisel et al., 2022 ), ihMT ( Girard et al., 2015 ), MTsat ( Helms et al., 2008 ))	Magnetization Transfer-weighted imaging	Myelin ( Morris et al., 2023 ; Soustelle et al., 2019 )	The more recently developed parameters, including MPF and ihMT, are highly specific to myelin.	The classic MTR derivation is somewhat non-specific, with sensitivity to water content and axonal density ( Hagiwara et al., 2018 ; Vavasour et al., 2011 ). More recent parameters have been largely restricted to the experimental imaging literature, with less clinical application
MWF	T2 relaxometry	Myelin ( Laule & Moore, 2018 )	Good dynamic range, and lower sensitivity to axonal directionality ( Morris et al., 2023 ).	Has a worse correlation with ground-truth myelin (as measured with myelin basic protein staining in a mouse model) than MPF ( Soustelle et al., 2019 ).
DTI parameters (FA, MD, RD, AxD)	Diffusion scan: minimum 1 b-value, six directions but preferably more ( Lebel et al., 2012 ; K. G. Schilling et al., 2017 )	Myelination, axonal integrity, somatic density, neurite density ( Galbusera et al., 2023 ; Gulani et al., 2001 ; Jones et al., 2013 )	Routine acquisition in research settings. One of the simplest diffusion acquisition paradigms, making it appealing for clinical applications. Extensive literature for comparison.	Non-specific, both in terms of histological correspondance and disease involvement.
Kurtosis	Diffusion scan (minimum 2 b-values of roughly 1000 and 2000)	Microstructural complexity ( Jensen et al., 2005 ; E. X. Wu & Cheung, 2010 )	Better specificity to structural changes in GM.	Non-specific to nature of structural change (e.g., axonal changes, somatic changes). Traditional derivations may not be robust ( Henriques et al., 2021 ).
NODDI	Diffusion scan: b-values of approximately 1000 and 2000 with 30–60 directions each ( H. Zhang et al., 2012 )	Intra-neurite, extra-neurite, and free water compartments ( H. Zhang et al., 2012 )	Better specificity to somatic vs. neurite structure in complex tissue	Acquisition not available yet for many clinical contexts. Will not distinguish varying cell types with broadly similar structure (e.g., interneurons vs. glia)
SANDI	Diffusion scan: b-values of 1000, 3000, 5000, and 10,000, with 64–128 directions each ( Palombo et al., 2020 )	Intra-neurite, intra-somatic, extra-somatic, free water ( Palombo et al., 2020 )	Intra- and extra-somatic compartments allow quantification of cellular changes.	Highly involved acquisition is currently inaccessible to most studies, especially clinical.
Susceptibility-weighted parameters (R2*, χ , χ -separation ( Shin et al., 2021 ))	Susceptibility-weighted imaging/quantitative susceptibility mapping ( Ruetten et al., 2019 )	Myelin, iron	Well-validated, widely used acquisitions, with good specificity. Able to seperate iron and myelin (paramagnetic and diamagnetic strictly speaking) contributions to signal.	Not routinely acquired for clinical studies.
Proton Density	Proton density-weighted imaging ( Mezer et al., 2016 )	Apparent water concentration, inverse of lipid and macromolecular density	Well-validated, alternative measure of lipid density	Not routinely acquired for clinical studies.

**Table 4.** Table of abbreviations.

Abbreviation	Definition
ACT	anatomically constrained tractography
AxD	axial diffusivity
ASD	autism spectrum disorder
AD	Alzheimer’s disease
BOLD	blood oxygen level dependent
dMRI	diffusion magnetic resonance imaging
DTI	diffusion tensor imaging
DWM	deep white matter
FA	fractional anisotropy
FAT	frontal aslant tract
fMRI	functional magnetic resonance imaging
FOD	fibre orientation distribution
FTLD	fronto-temporal lobar degeneration
GMWMI	grey matter–white matter interface
MAF	medium association fibre
MD	mean diffusivity
MTR	magnetization transfer ratio
MWF	myelin water fraction
NADPHd	nicotinamide adenine dinucleotide phosphate diaphorase
NDI	neurite density index
NODDI	neurite orientation dispersion and density imaging
ODI	orientation dispersion index
PFC	pre-frontal cortex
QSM	quantitative susceptibility mapping
RD	radial diffusivity
SAF	short association fibre
SANDI	soma and neurite density imaging
SFG	superior frontal gyrus
SWM	superficial white matter
TBSS	tract-based spatial statistics
WMIN	white matter interstitial neuron

## 6 Conclusion

Investigation of the SWM thus far has given a reasonably complete understanding of its anatomy, especially of fascicles reproducible across subjects. We also have early prototypes for techniques to capture individual bundle variations; we expect these protocols to be a major focus of upcoming research. As the number of SWM atlases begins to proliferate, it will be important to find common reference systems for labelling and annotating SAFs (Wassermann et al., 2013). Thus far, atlas makers have generally annotated their SAFs based on the bundle termination points (Guevara et al., 2020); this has the advantage of clarity and easy extensibility. This convention is not used throughout the entire literature however (e.g., “Op_SF_0i” fromRomán et al. (2017)corresponds to part of the FAT). Researchers must thus be aware that atlas tracts may elsewhere be referred to by “irregular” names.

The gestational and neonatal development of the SWM has been extensively characterized with histological and neuroimaging analysis. Development across the rest of the lifespan is much more opaque. Several large imaging studies provide us with reference values for the basic diffusion parameters, including FA, MD, RD, and AxD (Pietrasik et al., 2023;K. G. Schilling, Archer, Yeh, et al., 2023). These values, however, are non-specific, giving little insight into cellular and molecular processes occurring across development. Deviation of these values, as seen in disease, also cannot be transparently interpreted.

Because of this, the use of basic imaging methods in exploratory studies isolated from any histological context is unlikely to yield productive knowledge. However, we do not hold such imaging, with its unmatched subject throughput, non-invasive applicability across human populations, and comparability to behavioural measures, to be without any merit whatsoever. Instead, we suggest neuroimaging should, as much as possible, be conducted and interpreted in the immediate context of*ex vivo*histological study. The work ofKirilina et al. (2020)exemplifies this approach: they used a combination of histochemical microscopy and*ex vivo*MRI to develop a model relating$R_{2}^{*}$to iron levels and myelin fraction in quantitative MRI from young, healthy controls. In another example, not related to the SWM,Galbusera et al. (2023)correlated diffusivity metrics with histologically stained myelin in multiple sclerosis lesions, finding a surprisingly high negative correlation between myelin levels and AxD. Such careful analysis should be conducted more generally, for instance, to confirm a link between reduced FA and increased WMIN density in schizophrenia, or to study the correspondence in AD between reduced FA and Aβplaques. This could transform standard, non-specific MR parameters into meaningful markers of disease phenotypes. Since many neuropsychiatric syndromes lack localized “lesions” but very likely involve a generalized neuronal functional deficit, a histologically informed understanding of SWM dysfunction holds a distinct promise of understanding their pathophysiology. A recent review byAlkemade et al. (2023)discusses this in more detail.

Finally, future work should attempt to clarify an operational definition for the SWM. Specific study of this layer is predicated on its unique structural and cellular characteristics, distinctions rendered meaningless if it cannot be reliably delineated. To fully account for the region’s developmental history and the confluence of cells and vessels it contains, the validity of operational definitions should be empirically tested using converging methods such as gene expression analysis, single-cell transcriptome analysis aimed at WMINs, and imaging studies focused on myelin, iron, and diffusivity (Jung & Kim, 2023). Since the width of the region may be narrower than common dMRI acquisitions (0.5–2 mm vs. the common 2 mm isotropic resolution), imaging studies should acknowledge this limitation and seek higher resolution whenever possible. The effect of resolution on SAF clustering should also be explored; the thickness of SAFs on a high-resolution image would provide a good heuristic for the resolution required for accurate SAF retrieval, especially for protocols sampling parameter values along the streamlines.

## Data Availability

No novel code or data was used in the production of this manuscript.
